# Therapeutic Potential of Platelet-Rich Plasma in Fracture Healing: A Comprehensive Review

**DOI:** 10.7759/cureus.62271

**Published:** 2024-06-12

**Authors:** Prathamesh Kale, Sandeep Shrivastava, Prashanth Balusani, Aditya Pundkar

**Affiliations:** 1 Orthopaedic Surgery, Jawaharlal Nehru Medical College, Datta Meghe Institute of Higher Education and Research, Wardha, IND

**Keywords:** mechanisms of action, clinical outcomes, therapeutic potential, bone regeneration, platelet-rich plasma (prp), fracture healing

## Abstract

Fracture healing is a dynamic process essential for the restoration of bone integrity and function. However, factors such as patient age, comorbidities, and the severity of the fracture can impede this process, leading to delayed healing or nonunion. Platelet-rich plasma (PRP) has emerged as a promising therapeutic option for enhancing fracture healing. PRP is an autologous blood product containing a concentrated mixture of platelets, growth factors, and cytokines known to promote tissue regeneration and repair. This comprehensive review provides an overview of the fracture healing process, emphasizing the importance of timely and efficient bone repair. We discuss the mechanisms underlying the purported efficacy of PRP in fracture healing, drawing upon both preclinical and clinical evidence. Preclinical studies in animal models have demonstrated the ability of PRP to accelerate fracture healing, stimulate osteogenesis, and enhance bone regeneration. Clinical studies have yielded mixed results, with some reporting positive outcomes in terms of accelerated healing and improved functional outcomes, while others have shown no significant benefits over standard treatments. Factors influencing the efficacy of PRP, such as timing of administration, PRP concentration, and patient-specific variables, are also examined. Furthermore, safety considerations and potential adverse effects associated with PRP therapy are discussed. Despite the promising preclinical findings, challenges remain in standardizing PRP formulations, optimizing administration protocols, and addressing unanswered questions regarding its long-term efficacy and safety. This review aims to provide insights into the therapeutic potential of PRP in fracture healing, informing future research directions and guiding clinical practice.

## Introduction and background

Fracture healing is a complex physiological process involving a series of sequential events aimed at restoring the structural integrity and function of bone tissue. This process typically includes an inflammatory phase, a reparative phase characterized by the formation of callus tissue, and a remodeling phase where the bone undergoes structural refinement [1]. Timely and efficient fracture healing is crucial for patients to regain mobility, prevent complications such as nonunion or malunion, and ultimately restore quality of life. However, factors such as age, comorbidities, and the severity of the fracture can impair the natural healing process, necessitating interventions to accelerate or augment bone repair [2].

Platelet-rich plasma (PRP) has emerged as a promising adjunctive therapy for enhancing fracture healing. PRP is derived from the patient’s own blood and contains a concentrated mixture of platelets, growth factors, and cytokines that are believed to facilitate tissue regeneration and repair processes. Its potential application in fracture management has generated considerable interest among clinicians and researchers alike [3]. The purpose of this comprehensive review is to critically evaluate the therapeutic potential of PRP in fracture healing. By synthesizing preclinical and clinical evidence, this review aims to elucidate the mechanisms of action of PRP, assess its efficacy in promoting fracture repair, explore factors influencing its effectiveness, and discuss safety considerations and future directions for research and clinical practice.

## Review

Mechanisms of fracture healing

Inflammatory Phase

The initial phase of fracture healing, known as the inflammatory phase, spans several days following the injury and is a crucial protective mechanism that kickstarts the healing process [4,5]. When a fracture occurs, blood vessels are disrupted, leading to a hematoma, or a mass of clotted blood, at the site of the bone break [4,5]. This hematoma acts as a scaffold for subsequent stages of bone healing [4]. Within 48 hours, chemotactic signaling mechanisms attract inflammatory cells, such as neutrophils and macrophages, to the fracture site [5]. These cells play pivotal roles in debris removal and the initiation of tissue repair [4,5]. The inflammatory stage entails the release of various chemical inflammatory mediators, including cytokines and growth factors [4,5]. These mediators recruit inflammatory cells and attract mesenchymal stem cells (MSCs) to kickstart the next stage while stimulating the differentiation of MSCs into chondroblasts and osteoblasts [4,5]. Disruptions to this finely orchestrated sequence of inflammatory events can impair fracture healing, as evidenced in animal models with deficiencies in key inflammatory cytokines such as interleukin (IL)-6 and tumor necrosis factor (TNF). Evidence suggests that inadequate biomechanical conditions within the fracture zone can also impact the early inflammatory phase and impede bone healing [6].

Repair Phase

During the repair phase of fracture healing, a pivotal stage unfolds, marked by the forming of both a soft callus and a hard callus, which collectively bridge the fracture gap and confer structural stability. Within this phase, MSCs transform into two distinct cell types: chondroblasts, responsible for synthesizing cartilage, and osteoblasts, tasked with generating bone tissue. MSCs orchestrate the creation of a soft callus comprising fibrocartilage and collagen, serving as a provisional scaffold to stabilize the fracture. Concurrently, granulation tissue, abundant in blood vessels and fibroblasts, develops, providing oxygen and essential nutrients to sustain bone healing [7]. As the repair phase advances, osteoblasts infiltrate the soft callus, initiating the deposition of fresh bone tissue and progressively converting the soft callus into a hard callus characterized by woven, immature bone. This transformative process, whereby cartilage transitions into bone, is termed endochondral ossification. The resultant hard callus spans the fracture gap, furnishing structural reinforcement crucial for bone healing. Typically extending over several weeks to months, the duration of the repair phase varies depending upon the severity and location of the fracture. This phase is paramount in restoring the bone’s mechanical integrity and sets the stage for the ensuing remodeling phase [8].

Remodeling Phase

The remodeling phase denotes the conclusive stage of fracture healing, wherein the nascent woven bone undergoes a transformative process to assume its mature, mechanically robust structure. This intricate process hinges on the synchronized activity of osteoclasts and osteoblasts. Osteoclasts assume a pivotal role during this phase by orchestrating the resorption of surplus bone tissue. These specialized bone cells break down and eliminate aged or compromised bone, facilitating the bone’s reshaping into its optimal configuration. Concurrently, osteoblasts engage in bone modeling, depositing fresh bone tissue. As the architects of new bone formation, the activity of osteoblasts bolsters and refines the bone’s structure [9]. The remodeling phase unfolds over an extended timeframe, often several years, as the bone gradually adapts to its demands. The bone undergoes reshaping and fortification throughout this duration to reclaim its preinjury morphology and functionality. Numerous factors can impede this remodeling phase, encompassing the nature and site of the fracture, the patient’s age, concurrent medical conditions, inadequate nutritional status, compromised blood circulation, and infections. Comprehending the mechanisms underpinning the remodeling phase is paramount in optimizing fracture healing outcomes and ensuring optimal patient recovery [10]. By elucidating these intricate processes, clinicians can tailor treatment strategies to facilitate efficient bone remodeling and enhance patient outcomes following fracture injury.

Platelet-rich plasma

Composition and Preparation Methods

PRP is a regenerative therapy approach involving the creation of a concentrated solution of autologous platelets suspended within a small plasma volume. Various techniques exist for PRP preparation, with the PRP method and the buffy-coat method ranking among the most prevalent. These methodologies yield distinct PRP products, including pure PRP (P-PRP), leucocyte PRP (L-PRP), pure platelet-rich fibrin (P-PRF), and leucocyte PRF (L-PRF) [11]. Typically, the PRP preparation process involves collecting a patient’s blood at the treatment juncture and then segregate it into constituent components leveraging a specialized centrifuge apparatus. This segregation, achieved through differential centrifugation, encompasses an initial phase aimed at separating red blood cells, succeeded by a subsequent centrifugation step to concentrate platelets. Utilizing a patient’s blood for PRP preparation often proves more cost-effective than commercial kits [12]. The composition of PRP exhibits variability contingent upon the specific preparation protocol employed, resulting in diverse products such as platelet-poor plasma (PPP) and platelet lysate (PL). Rich in platelets, PRP harbors growth factors that are instrumental in facilitating tissue repair and healing. Tailoring the concentration of platelets and white blood cells (WBCs) and incorporating additives can align with particular therapeutic objectives. Nevertheless, the absence of standardized guidelines engenders disparities in preparation methodologies across practitioners [11]. The Indian Association of Dermatologists, Venereologists and Leprologists has proffered recommendations concerning PRP preparation. Advocating for a double-spin manual method, they stipulate specific centrifuge speed parameters and platelet concentration thresholds tailored to various dermatological conditions. Moreover, they underscore that PRP activation proves unnecessary when administering injections into soft tissues, emphasizing the pivotal role of the preparation methodology in dictating PRP treatment efficacy [12].

Biological Components and Functions

PRP is a biological derivative extracted from a patient’s blood, boasting an elevated concentration of platelets and growth factors. This biological concoction comprises several key components, including platelets, WBCs, growth factors, cytokines, proteins, and enzymes [13]. Platelets, renowned for their pivotal role in the healing cascade, discharge many bioactive factors upon activation, thereby instigating tissue repair and regeneration. With over 1,100 distinct proteins, including growth factors, enzymes, and cytokines, platelets orchestrate crucial processes such as inflammation modulation, angiogenesis promotion, and cell proliferation [14]. Moreover, PRP houses WBCs, notably leukocytes, which safeguard the body against infection and orchestrate the inflammatory response. Various subclasses of WBCs, including progenitor cells, harbor unique biological functionalities, ranging from facilitating angiogenesis to differentiating into diverse cell types [15]. A plethora of growth factors pervades PRP, encompassing vascular endothelial growth factor (VEGF), fibroblast growth factor (FGF), platelet-derived growth factor (PDGF), epidermal growth factor (EGF), and insulin-like growth factors (IGF-1 and IGF-2). These growth factors, liberated by platelets, drive pivotal biological processes such as angiogenesis induction, inflammation resolution, and tissue regeneration [16]. Furthermore, as signaling molecules, cytokines influence PRP by modulating the immune response, thereby impacting the healing trajectory. Additionally, an array of proteins and enzymes in PRP partakes in diverse biological processes, spanning coagulation, inflammation modulation, and tissue repair [17]. The biological functionalities encapsulated within PRP encompass hemostasis, inflammation and immune response modulation, angiogenesis promotion, tissue regeneration, and wound healing facilitation. The amalgamation of platelets, growth factors, cytokines, proteins, and enzymes within PRP orchestrates an optimal milieu conducive to tissue healing and regeneration, rendering it a valuable therapeutic modality across various medical applications [18].

Mechanisms of Action in Fracture Healing

**Figure 1:**
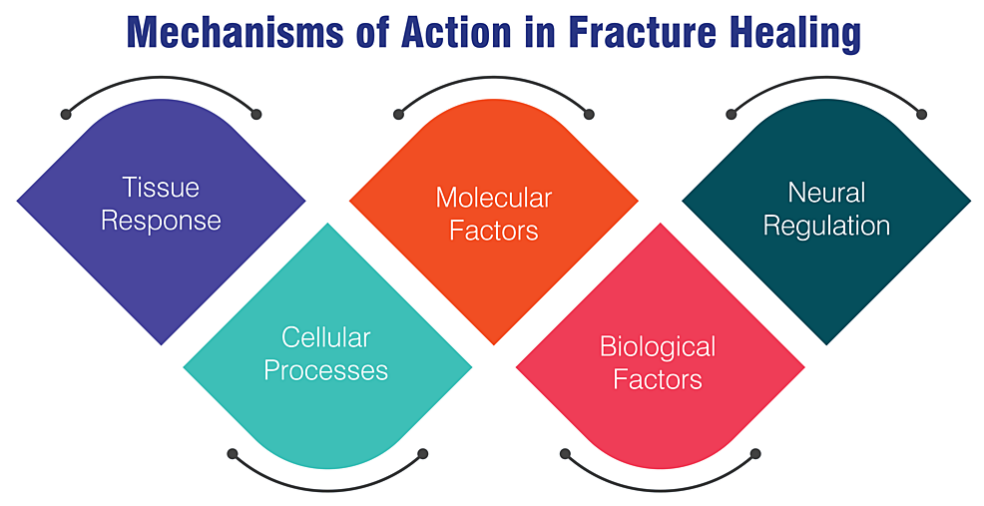
Mechanisms of action in fracture healing. Image credit: Dr. Prathamesh Kale.

Fracture healing commences with an anabolic phase characterized by tissue response, wherein local tissue volume expands via inflammation, culminating in the formation of a hematoma following fracture occurrence [19]. Subsequently, a cascade of cellular processes ensues, encompassing cell migration, tissue differentiation, synthesis, and the release of cytokines and growth factors, all intricately regulated by the mechanical microenvironment [19]. The healing trajectory involves pivotal biological factors such as fibroblasts, chondroblasts, and osteoblasts, with the repair phase hallmarking the differentiation of versatile mesenchymal cells into these specialized cell types [20]. In addition to cellular dynamics, neural regulation emerges as a critical determinant in fracture repair, exerting influence over pain management, swelling, and bone recovery. Peripheral sensory nerves contribute to the healing process by releasing neuropeptides that augment repair, while the involvement of the central nervous system can both expedite and impede healing outcomes [20]. Molecular factors, including neuropeptides (NPY, CGRP), growth factors (EGF, NGF), and neurotransmitters, further contribute to bone homeostasis, angiogenesis, and neuronal development, thereby impacting fracture healing [20]. Comprehending these multifaceted mechanisms assumes paramount importance in the formulation of effective strategies to mitigate complications such as delayed union, nonunion, and post-traumatic fracture pain. Continued exploration into the neural regulation of fracture healing holds promise for developing targeted therapies to enhance healing processes and pain management, ultimately fostering improved patient outcomes post-fracture [20]. Mechanisms of action in fracture healing are shown in Figure 1.

Preclinical studies on platelet-rich plasma in fracture healing

Animal Models Used

Animal models have been extensively employed to investigate the impact of PRP on fracture healing. Preclinical investigations utilizing various animal long-bone fracture models have consistently reported favorable outcomes associated with PRP administration, including accelerated callus formation, heightened bone mineral density, and enhanced biomechanical properties at the healing fracture site compared to control groups [21-23]. The predominant treatment modality in animal studies involved fixation surgery concomitant with localized PRP injection at the fracture site [22,23]. Rabbits and rodents emerged as the most prevalent animal models utilized for assessing the efficacy of PRP in bone healing, with research conducted in rabbit models featuring critical-sized bone defects and rat models replicating femoral and tibial fractures [22]. Moreover, animal investigations have underscored the capacity of PRP to stimulate the proliferation and differentiation of osteoblast-like cells in vitro, hinting at its potential to foster bone regeneration [21,23]. Nevertheless, the translatability of these promising preclinical findings to clinical settings remains incomplete. Despite observations indicating that PRP administration can abbreviate fracture healing duration in patients, its ability to consistently enhance the overall fracture healing rate vis-à-vis standard treatments alone remains equivocal [21,23].

Findings Regarding Fracture Healing Improvement

Experimental research has elucidated that the size of the fracture gap significantly influences the pace of bone healing, with wider gaps often resulting in delayed healing and critical-size defects posing challenges for sufficient restoration [24]. Additionally, factors such as muscle trauma, intraoperative trauma, and interference with the healing process by surgical interventions such as hematoma or periosteum removal can impede the natural course of healing [24]. Fracture healing comprises several pivotal stages, including hematoma formation, granulation tissue formation, bony callus formation, and bone remodeling. The type of bone healing, whether primary or secondary, is intricately linked to the mechanical stability attained at the fracture site. Primary bone healing occurs under conditions of mechanical strain below 2%, whereas secondary bone healing transpires within a strain range of 2% to 10%. Excessive mechanical strain exceeding 10% can predispose to nonunion or delayed union [1]. A spectrum of methodologies has been devised to augment fracture healing, encompassing autografts, allografts, ultrasound therapy, and autologous cultured osteoblasts. These approaches aim to expedite union via osteogenesis, osteoconduction, and osteoinduction. Nonetheless, each method presents distinct advantages and drawbacks, including concerns over donor-site morbidity associated with autografts and the potential for immune reactions with allografts [25].

Clinical studies on platelet-rich plasma in fracture healing

Study Designs and Methodologies

A systematic review comprising 26 preclinical studies and nine clinical trials scrutinized the efficacy of PRP in fracture healing, revealing that while PRP shortened the duration of bony healing, it failed to yield positive outcomes in enhancing the healing rate of closed fractures. The findings exhibited heterogeneity attributed to divergent study protocols and fracture types [26]. Encouragingly, certain investigations showcased that PRP bolstered the healing rate of nonunion fractures in both animal models and human subjects, suggesting its potential to stimulate the healing cascade [23]. Despite the promising indications, a clinical study indicated that while PRP hastened fracture healing time, it did not improve the overall healing rate compared to standard treatments [23,26]. Notably, the preparation and administration protocols of PRP varied across studies, contributing to the observed inconsistencies. Discrepancies included divergent platelet counts and the absence of a standardized application technique [23]. The systematic review underscored several limitations, notably the lack of standardization in PRP preparation methodologies, the marked heterogeneity among enrolled studies, and the imperative for further exploration into PRP characteristics such as platelet concentration and leukocyte numbers [26]. To elucidate the precise role of PRP in fracture healing, rigorous, long-term clinical trials of high caliber are imperative. Such trials should delve into optimal PRP formulation, the timing of administration, and judicious patient selection criteria [23,26].

Patient Populations Studied

Clinical studies investigating the efficacy of PRP in fracture healing have predominantly centered on patients with traumatic fractures, with a subset of studies also encompassing pathological or periprosthetic fractures. The bulk of enrolled patients in these clinical investigations presented with traumatic fractures, including closed fractures of long bones, mandibular fractures, and nonunion fractures [23,26,27]. Specific examinations delved into the application of PRP in patients with atrophic nonunion fractures post-internal fixation of ulnar fractures and those with mandibular fractures managed with PRP in conjunction with standard fixation [26,28]. The primary body of clinical evidence regarding PRP for fracture healing comprises case series and observational studies, with a noticeable dearth of high-quality randomized controlled trials [26,27]. The variability in fracture types and PRP preparation protocols across these studies complicates the derivation of definitive conclusions [23,26]. While PRP is generally deemed a safe adjunct therapy, its clinical efficacy in enhancing fracture healing rates remains controversial. While certain investigations have reported a reduction in fracture healing duration with PRP, the consistent improvement in overall healing rates compared to standard treatments alone remains elusive [23,26,27]. In essence, clinical studies investigating PRP for fracture healing predominantly involve patients with traumatic fractures, with varied outcomes regarding its ability to expedite healing and enhance outcomes. The imperative for more standardized, high-quality trials persists to elucidate the precise role of PRP in fracture management across diverse patient populations.

Outcomes and Efficacy Assessments

Clinical studies have provided evidence that PRP can abbreviate the duration of fracture healing time. However, its impact on the overall fracture healing rate compared to standard treatments has been inconsistent [23,26]. While some studies have suggested that PRP may facilitate the healing of nonunion fractures in select cases, the overall effects of PRP on functional outcomes and fracture healing rates have generated controversy [28]. The variability in PRP preparation protocols and the diversity of fractures studied likely contribute to the disparate clinical results observed. Further investigation into factors such as optimal PRP formulation, timing of administration, and patient selection criteria is warranted. Despite its reputation as a safe and straightforward therapy, establishing the definitive role of PRP in fracture management necessitates more high-quality, long-term clinical trials [23,26]. Presently, the available evidence hints at the potential of PRP as a supplementary treatment for fractures. However, its precise clinical benefits remain to be fully elucidated, emphasizing the need for continued research in this domain.

Factors influencing the efficacy of platelet-rich plasma in fracture healing

Timing of Platelet-Rich Plasma Administration

Administering PRP promptly, ideally within three days following a fracture event, is pivotal for maximizing its beneficial impact on bone regeneration and minimizing the likelihood of revision surgery [29,30]. During the initial 0-3 days post-procedure, it is recommended to ensure the complete rest of the treated area, allowing for proper absorption of platelets into the joint [29]. Utilization of immobilization devices such as slings or walking boots may be necessary to limit the use of the affected limb during this phase [29]. From days 3-14, gradual reintroduction of light workouts such as basic yoga and weight-bearing exercises can be initiated as tolerated while refraining from activities that impose compression or strain on the injected joint [29]. Subsequently, after 14 days, more intensive exercises such as lifting, stretching, and cardiovascular activities can be gradually incorporated under careful guidance from the medical provider. The intensity of these activities should be progressively increased to facilitate the continued action of PRP while regaining strength [29]. Patients typically resume full sports and activities around 6-9 weeks following PRP injections upon clearance from the provider [29,30]. It is common to experience some residual soreness, which typically resolves within 24 hours [29]. Timing of PRP administration is critical, as early injection within the initial days optimizes its efficacy in fracture healing. At the same time, premature resumption of activity can disrupt PRP and diminish its effectiveness [29,30]. Adhering closely to the prescribed recovery timeline is essential for maximizing the benefits of PRP treatment.

Platelet-Rich Plasma Concentration and Composition

Different PRP preparation methods yield significant differences in leukocyte concentration, ranging from 14.9 ± 4.5 (10^3^/μL) in leukocyte-rich PRP (LR-PRP) to 0.2 ± 0.2 (10^3^/μL) in pure-PRP, while platelet concentration remains relatively consistent across methods [31]. The presence of leukocytes strongly influences PRP quality by impacting the concentrations of growth factors and proteases. Notably, leukocyte concentration positively correlates with PDGF-BB and VEGF levels but inversely correlates with FGF-b. Additionally, it exhibits a strong positive correlation with matrix metalloproteinase-9 concentration [31]. Baseline platelet count exhibits a directly proportional relationship with PRP platelet count, with a 3.8× increase observed for each unit rise in baseline blood platelet count [32]. Moreover, age inversely impacts PRP platelet count, with an approximate decrease of 32,666 platelets in the final PRP product noted for every decade increase in age [32]. Intrapersonal variability is also evident, as the second dose of PRP administered to the same patient demonstrates a significantly higher mean platelet count than the initial dose, with a mean difference of 354,448 [32]. However, demographic factors such as sex, body mass index, and other components of baseline blood count (leukocytes, lymphocytes, neutrophils) do not significantly influence the final PRP composition [32,33]. These findings underscore the substantial variability in PRP composition attributable to patient demographics and preparation methods, which may contribute to the inconsistent clinical outcomes observed in PRP studies. Consequently, standardization of PRP preparation protocols and consideration of patient-specific factors is imperative to optimize PRP therapy.

Patient Factors

Various patient-related factors can influence the efficacy of PRP therapy in fracture healing. Aging is associated with lower platelet counts and diminished growth factor concentrations in PRP, potentially impacting its effectiveness [23,34]. Moreover, gender differences in the composition and concentration of growth factors in PRP have been suggested, which could affect healing outcomes [23]. Platelet count in PRP plays a critical role, with higher platelet counts associated with elevated growth factor levels and potentially improved fracture healing. However, the optimal platelet concentration remains a subject of ongoing investigation [23,34]. Furthermore, the type of fracture may influence the efficacy of PRP treatment, with particular benefits observed for mandibular fractures and nonunions. The location and severity of the fracture can also influence the response to PRP therapy [34,35]. Underlying medical conditions such as diabetes, smoking, and osteoporosis may negatively impact fracture healing and diminish the effectiveness of PRP [27,34]. The stability of fracture fixation is another crucial consideration, as stable fractures with rigid internal fixation may derive greater benefits from PRP, given its ability to enhance biological healing aspects in an optimized biomechanical environment [27,35]. Additionally, the timing of PRP administration is paramount, with early intervention within the first few weeks after fracture potentially more effective in promoting bone regeneration and reducing the risk of delayed union or nonunion [34,35]. These patient-specific factors underscore the importance of individualized treatment approaches and careful consideration in applying PRP therapy for fracture management.

Safety considerations and adverse effects of platelet-rich plasma in fracture healing

Risk of Infection

Infection represents a potential but rare side effect of PRP injections [36-38]. Ensuring proper sterilization techniques and maintaining a clean environment are essential to mitigate this risk [37,38]. For instance, a study involving 1,073 patients who underwent PRP injections for rotator cuff injuries reported only two cases of infection, both effectively treated with antibiotics [38]. Additionally, it is noteworthy that patients with chronic urticaria (hives) experienced exacerbation of symptoms following PRP injections. However, this was likely attributed to their underlying condition rather than the PRP [38]. To minimize the risk of infection, meticulous disinfection of the skin surrounding the injection site should be performed before inserting the needle. Subsequently, after each injection, the site should be covered with a clean cotton ball, and pressure should be applied [37]. Opting for a qualified and experienced healthcare provider who adheres to proper sterile techniques is imperative in reducing the likelihood of infection associated with PRP injections [38].

Potential for Adverse Reactions

PRP therapy is generally considered safe for fracture healing, with rare adverse events documented in the literature. Derived from the patient’s own blood, PRP minimizes the risk of allergic reactions or immunological responses. Studies have demonstrated that PRP injections do not elevate the incidence of postoperative wound infections compared to control groups, further bolstering its safety profile in fracture management [39]. Although adverse reactions associated with PRP are infrequent, the variability in clinical outcomes is often attributed to the lack of standardization in PRP preparation protocols and application techniques. Standardizing these procedures is imperative to ensure consistent efficacy and safety in fracture healing. Furthermore, determining the optimal formulation of PRP, including considerations such as platelet concentration and leukocyte numbers, necessitates further investigation to ascertain the most effective and safe utilization of PRP in fracture treatment [40]. While PRP has demonstrated potential in expediting fracture healing in certain instances, its overall impact on fracture healing rates vis-à-vis standard treatments remains inconclusive. The mixed findings regarding functional outcomes underscore the necessity for additional research to gain deeper insights into the therapeutic potential of PRP in fracture management. Despite these considerations, PRP therapy continues to be explored as a safe and potentially beneficial adjunctive treatment for fractures, with ongoing endeavors to refine its application and optimize its clinical benefits [41].

Long-Term Effects

The clinical evidence concerning the effectiveness of PRP for fracture healing presents a mixed picture. While certain studies indicate that PRP can reduce fracture healing time, it does not consistently enhance the overall fracture healing rate compared to standard treatments [34,42]. A meta-analysis revealed that PRP adjunct therapy shortened fracture healing duration and improved bone mineral density in certain cases, particularly in mandibular fractures [34]. However, the analysis did not assess long-term outcomes beyond the study periods. Thus, further investigation into factors such as optimal PRP formulation, timing of administration, and patient selection criteria is necessary to ascertain the true long-term effects of PRP on fracture healing [34,42]. The lack of standardization in PRP preparation protocols contributes to the variability in clinical results. While PRP is generally regarded as a safe treatment, with rare adverse events reported, the long-term safety profile remains incompletely established [34,42]. Consequently, longer-term randomized clinical trials are warranted to gain a deeper understanding of the enduring therapeutic potential and risks associated with PRP utilization in fracture healing.

## Conclusions

This comprehensive review underscores the promising role of PRP in enhancing fracture healing. Through synthesizing preclinical and clinical evidence, it becomes evident that PRP offers a viable adjunctive therapy for accelerating and augmenting the natural process of bone repair. While preclinical studies have provided valuable insights into how PRP promotes fracture healing, clinical investigations have demonstrated its potential clinical utility in improving fracture union rates and expediting patient recovery.
